# 2-[(1,3-Benzothia­zol-2-yl)imino­meth­yl]phenol

**DOI:** 10.1107/S1600536809007934

**Published:** 2009-03-11

**Authors:** Si-Quan Liu, Cai-Feng Bi, Liang-Yu Chen, Yu-Hua Fan

**Affiliations:** a School of Chemistry and Chemical Engineering, Ocean University of China, Qingdao 266012, People’s Republic of China; b School of Chemistry and Chemical Engineering, University of Jinan, Jinan 250022, People’s Republic of China

## Abstract

The title compound, C_14_H_10_N_2_OS, is nearly planar, with a maximum deviation of 0.0698 (13) Å from the mean plane, and exists in an *E* configuration with respect to the C=N bond. The dihedral angle between the two benzene rings is 2.81 (9)°. There is an intra­molecular O—H⋯N hydrogen bond and inter­molecular C—H⋯O and C—H⋯N hydrogen bonds.

## Related literature

For related structures of 2-amino­benzothia­zole derivatives and their Schiff bases, see: Büyükgüngör *et al.* (2004 ▶); Liang *et al.* (1999 ▶); Liu *et al.* (2009 ▶). For the biological activity of the title compound and related structures, see: Yan *et al.* (1999 ▶).
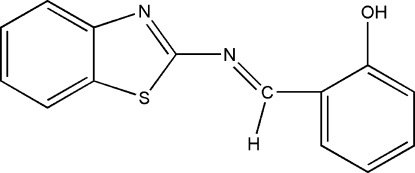


## Experimental

### 

#### Crystal data

C_14_H_10_N_2_OS
*M* *_r_* = 254.30
Orthorhombic, 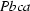

*a* = 12.150 (2) Å
*b* = 8.9578 (15) Å
*c* = 22.026 (4) Å
*V* = 2397.4 (7) Å^3^
*Z* = 8
Mo *K*α radiation
μ = 0.26 mm^−1^
*T* = 298 K
0.51 × 0.15 × 0.11 mm

#### Data collection

Bruker SMART APEX diffractometer
Absorption correction: none
12166 measured reflections
2353 independent reflections
1939 reflections with *I* > 2σ(*I*)
*R*_int_ = 0.036

#### Refinement

*R*[*F* ^2^ > 2σ(*F* ^2^)] = 0.045
*wR*(*F* ^2^) = 0.113
*S* = 1.06
2353 reflections
163 parameters
H-atom parameters constrained
Δρ_max_ = 0.26 e Å^−3^
Δρ_min_ = −0.24 e Å^−3^

### 

Data collection: *SMART* (Bruker, 2000 ▶); cell refinement: *SAINT* (Bruker, 2000 ▶); data reduction: *SAINT*; program(s) used to solve structure: *SHELXS97* (Sheldrick, 2008 ▶); program(s) used to refine structure: *SHELXL97* (Sheldrick, 2008 ▶); molecular graphics: *SHELXTL* (Sheldrick, 2008 ▶); software used to prepare material for publication: *SHELXTL*.

## Supplementary Material

Crystal structure: contains datablocks I, global. DOI: 10.1107/S1600536809007934/is2387sup1.cif

Structure factors: contains datablocks I. DOI: 10.1107/S1600536809007934/is2387Isup2.hkl

Additional supplementary materials:  crystallographic information; 3D view; checkCIF report

## Figures and Tables

**Table 1:** Hydrogen-bond geometry (Å, °)

*D*—H⋯*A*	*D*—H	H⋯*A*	*D*⋯*A*	*D*—H⋯*A*
O1—H1⋯N2	0.82	1.88	2.6034 (19)	147
C7—H7⋯O1^i^	0.93	2.43	3.309 (2)	158
C2—H2⋯N1^ii^	0.93	2.68	3.593 (2)	167

Symmetry codes: (i) 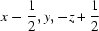; (ii) 
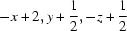
.

**Fig. 1.**
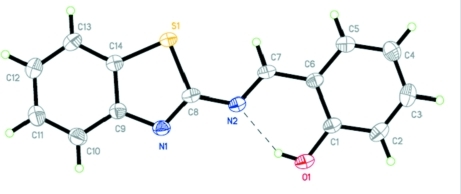
Molecular structure of the title compound, with 30% probability displacement ellipsoids.

**Fig. 2.**
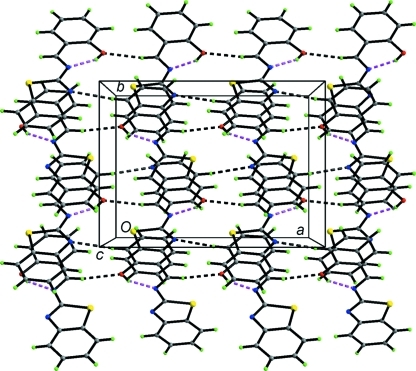
A partial packing diagram of the title compound. Hydrogen bonds were shown by dashed lines.

## supplementary crystallographic information

### Comment

A wide range of biological activities have been attributed to the title compounds and compound having similar structure (Yan *et al.*, 1999). One kind of schiff base of 2-aminobenzothiazole was prepared by Büyükgüngör *et al.* (2004). The title compound has been prepared to utilize it as an intermediate ligand and for complexation with various metals (Liang *et al.*, 1999; Liu *et al.*, 2009).

In the molecule of the title compound (Fig. 1), the bond length of C8—N2 [1.379 (2) Å] is shorter than normal C—N (1.47 Å). The entire molecule is almost planar due to the C6—C7—N2—C8—N1—C9 π-π conjunction. The dihedral angle between the two benzene rings (C1—C6 and C9—C14) is 2.81 (9)°. The benzothiazol and the *o*-hydroxy benzenyl at the C=N double bond are in an E configuration due to the hydrogen bond between O—H···N.

In the crystal structure, intermolecular C—H···O and C—H···N hydrogen bonds (Table 1) link the molecules (Fig. 2), in which they may be effective in the stabilization of the structure.

### Experimental

2-Aminobenzithiazole (0.01 mol) and salicylaldehyde (0.01 mol) were dissolved in 50 ml ethanol at 298 K, then the reaction temperature raised to 343 K. After 3 h of reaction, the reaction mixture was condensed to 20 ml and cooled down to 273 K to give a dark orange solid. The crude was purified by column chromatography, affording salmon pink crystals of the title compound (yield 91%; m.p. 417–418 K).

### Refinement

H atoms were positioned geometrically (O—H = 0.82 Å for OH, C—H = 0.93 Å for aromatic H and C—H = 0.93 Å for acyclic H) and were refined as riding, with *U*_iso_(H) = 1.5*U*_eq_(O) or 1.2*U*_eq_(C).

### Crystal data

**Table:**

C_14_H_10_N_2_OS	*D*_x_ = 1.409 Mg m^−^^3^
*M**_r_* = 254.30	Melting point: 417 K
Orthorhombic, *P**b**c**a*	Mo *K*α radiation, λ = 0.71073 Å
Hall symbol: -P 2ac 2ab	Cell parameters from 3147 reflections
*a* = 12.150 (2) Å	θ = 2.5–27.1°
*b* = 8.9578 (15) Å	µ = 0.26 mm^−^^1^
*c* = 22.026 (4) Å	*T* = 298 K
*V* = 2397.4 (7) Å^3^	Rod, yellow
*Z* = 8	0.51 × 0.15 × 0.11 mm
*F*(000) = 1056	

### Data collection

**Table:**

Bruker SMART APEX diffractometer	1939 reflections with *I* > 2σ(*I*)
Radiation source: fine-focus sealed tube	*R*_int_ = 0.036
graphite	θ_max_ = 26.0°, θ_min_ = 1.9°
φ and ω scans	*h* = −14→14
12166 measured reflections	*k* = −11→10
2353 independent reflections	*l* = −27→18

### Refinement

**Table:**

Refinement on *F*^2^	Primary atom site location: structure-invariant direct methods
Least-squares matrix: full	Secondary atom site location: difference Fourier map
*R*[*F*^2^ > 2σ(*F*^2^)] = 0.045	Hydrogen site location: inferred from neighbouring sites
*wR*(*F*^2^) = 0.113	H-atom parameters constrained
*S* = 1.06	*w* = 1/[σ^2^(*F*_o_^2^) + (0.0596*P*)^2^ + 0.4758*P*] where *P* = (*F*_o_^2^ + 2*F*_c_^2^)/3
2353 reflections	(Δ/σ)_max_ = 0.001
163 parameters	Δρ_max_ = 0.26 e Å^−^^3^
0 restraints	Δρ_min_ = −0.24 e Å^−^^3^

### Special details

**Table:**

Geometry. All e.s.d.'s (except the e.s.d. in the dihedral angle between two l.s. planes) are estimated using the full covariance matrix. The cell e.s.d.'s are taken into account individually in the estimation of e.s.d.'s in distances, angles and torsion angles; correlations between e.s.d.'s in cell parameters are only used when they are defined by crystal symmetry. An approximate (isotropic) treatment of cell e.s.d.'s is used for estimating e.s.d.'s involving l.s. planes.
Refinement. Refinement of *F*^2^ against ALL reflections. The weighted *R*-factor *wR* and goodness of fit *S* are based on *F*^2^, conventional *R*-factors *R* are based on *F*, with *F* set to zero for negative *F*^2^. The threshold expression of *F*^2^ > σ(*F*^2^) is used only for calculating *R*-factors(gt) *etc*. and is not relevant to the choice of reflections for refinement. *R*-factors based on *F*^2^ are statistically about twice as large as those based on *F*, and *R*- factors based on ALL data will be even larger.

### Fractional atomic coordinates and isotropic or equivalent isotropic displacement parameters (Å^2^)

**Table:**

	*x*	*y*	*z*	*U*_iso_*/*U*_eq_	
C1	0.88027 (14)	0.3483 (2)	0.21383 (9)	0.0409 (4)	
C2	0.91856 (18)	0.4419 (2)	0.16848 (10)	0.0541 (6)	
H2	0.9937	0.4497	0.1611	0.065*	
C3	0.8451 (2)	0.5230 (3)	0.13448 (10)	0.0570 (6)	
H3	0.8715	0.5858	0.1042	0.068*	
C4	0.73328 (19)	0.5138 (2)	0.14422 (9)	0.0530 (5)	
H4	0.6847	0.5691	0.1206	0.064*	
C5	0.69463 (16)	0.4221 (2)	0.18923 (9)	0.0451 (5)	
H5	0.6192	0.4159	0.1960	0.054*	
C6	0.76636 (14)	0.3374 (2)	0.22515 (8)	0.0370 (4)	
C7	0.72317 (13)	0.2428 (2)	0.27182 (8)	0.0381 (4)	
H7	0.6474	0.2382	0.2773	0.046*	
C8	0.74236 (14)	0.0726 (2)	0.35054 (8)	0.0366 (4)	
C9	0.74360 (15)	−0.0870 (2)	0.42653 (8)	0.0397 (4)	
C10	0.78616 (17)	−0.1805 (2)	0.47105 (9)	0.0519 (5)	
H10	0.8619	−0.1912	0.4753	0.062*	
C11	0.71662 (19)	−0.2563 (3)	0.50844 (10)	0.0574 (6)	
H11	0.7452	−0.3190	0.5382	0.069*	
C12	0.60333 (19)	−0.2410 (3)	0.50247 (11)	0.0614 (6)	
H12	0.5570	−0.2935	0.5284	0.074*	
C13	0.55866 (18)	−0.1495 (3)	0.45897 (10)	0.0589 (6)	
H13	0.4828	−0.1395	0.4551	0.071*	
C14	0.62954 (15)	−0.0724 (2)	0.42097 (9)	0.0419 (4)	
N1	0.80522 (12)	−0.00477 (18)	0.38564 (7)	0.0419 (4)	
N2	0.78539 (11)	0.16428 (17)	0.30618 (7)	0.0382 (4)	
O1	0.95340 (10)	0.26804 (18)	0.24619 (7)	0.0549 (4)	
H1	0.9210	0.2214	0.2728	0.082*	
S1	0.59914 (4)	0.05133 (6)	0.36285 (2)	0.04566 (19)	

### Atomic displacement parameters (Å^2^)

**Table:**

	*U*^11^	*U*^22^	*U*^33^	*U*^12^	*U*^13^	*U*^23^
C1	0.0337 (9)	0.0443 (11)	0.0447 (10)	−0.0016 (8)	0.0039 (8)	−0.0077 (8)
C2	0.0457 (11)	0.0596 (14)	0.0571 (13)	−0.0105 (10)	0.0130 (10)	−0.0046 (10)
C3	0.0714 (16)	0.0517 (13)	0.0480 (12)	−0.0107 (12)	0.0098 (11)	0.0030 (10)
C4	0.0639 (14)	0.0503 (12)	0.0449 (12)	0.0039 (11)	−0.0043 (10)	0.0009 (9)
C5	0.0394 (10)	0.0477 (12)	0.0481 (11)	0.0018 (9)	−0.0018 (8)	−0.0045 (9)
C6	0.0320 (9)	0.0384 (10)	0.0405 (10)	−0.0008 (7)	0.0018 (7)	−0.0081 (8)
C7	0.0261 (8)	0.0428 (10)	0.0455 (10)	−0.0003 (8)	0.0018 (7)	−0.0058 (8)
C8	0.0277 (9)	0.0405 (10)	0.0416 (10)	0.0008 (7)	0.0012 (7)	−0.0073 (8)
C9	0.0390 (10)	0.0424 (11)	0.0378 (10)	0.0018 (8)	0.0022 (8)	−0.0064 (8)
C10	0.0478 (11)	0.0582 (13)	0.0497 (12)	0.0082 (10)	−0.0029 (9)	0.0003 (10)
C11	0.0681 (14)	0.0566 (14)	0.0475 (12)	0.0035 (11)	−0.0003 (10)	0.0086 (10)
C12	0.0591 (14)	0.0670 (15)	0.0579 (14)	−0.0063 (12)	0.0101 (10)	0.0144 (12)
C13	0.0427 (11)	0.0685 (15)	0.0656 (14)	−0.0061 (10)	0.0086 (10)	0.0116 (12)
C14	0.0384 (9)	0.0430 (11)	0.0443 (11)	−0.0005 (8)	0.0002 (8)	−0.0022 (8)
N1	0.0332 (8)	0.0472 (9)	0.0453 (9)	0.0050 (7)	0.0017 (7)	−0.0014 (8)
N2	0.0302 (7)	0.0420 (9)	0.0425 (8)	0.0007 (6)	0.0034 (6)	−0.0034 (7)
O1	0.0289 (6)	0.0699 (10)	0.0660 (10)	0.0016 (7)	0.0052 (6)	0.0088 (8)
S1	0.0277 (3)	0.0558 (4)	0.0535 (3)	−0.0020 (2)	−0.00019 (19)	0.0079 (2)

### Geometric parameters (Å, °)

**Table:**

C1—O1	1.347 (2)	C8—N2	1.379 (2)
C1—C2	1.384 (3)	C8—S1	1.7714 (18)
C1—C6	1.410 (2)	C9—N1	1.384 (2)
C2—C3	1.373 (3)	C9—C10	1.389 (3)
C2—H2	0.9300	C9—C14	1.397 (3)
C3—C4	1.378 (3)	C10—C11	1.362 (3)
C3—H3	0.9300	C10—H10	0.9300
C4—C5	1.371 (3)	C11—C12	1.390 (3)
C4—H4	0.9300	C11—H11	0.9300
C5—C6	1.400 (3)	C12—C13	1.373 (3)
C5—H5	0.9300	C12—H12	0.9300
C6—C7	1.432 (3)	C13—C14	1.385 (3)
C7—N2	1.280 (2)	C13—H13	0.9300
C7—H7	0.9300	C14—S1	1.733 (2)
C8—N1	1.289 (2)	O1—H1	0.8200
			
O1—C1—C2	118.89 (16)	N2—C8—S1	123.03 (13)
O1—C1—C6	121.15 (17)	N1—C9—C10	125.37 (17)
C2—C1—C6	119.96 (18)	N1—C9—C14	115.45 (16)
C3—C2—C1	119.71 (19)	C10—C9—C14	119.17 (18)
C3—C2—H2	120.1	C11—C10—C9	119.79 (19)
C1—C2—H2	120.1	C11—C10—H10	120.1
C2—C3—C4	121.6 (2)	C9—C10—H10	120.1
C2—C3—H3	119.2	C10—C11—C12	120.5 (2)
C4—C3—H3	119.2	C10—C11—H11	119.7
C5—C4—C3	119.1 (2)	C12—C11—H11	119.7
C5—C4—H4	120.4	C13—C12—C11	121.1 (2)
C3—C4—H4	120.4	C13—C12—H12	119.4
C4—C5—C6	121.34 (19)	C11—C12—H12	119.4
C4—C5—H5	119.3	C12—C13—C14	118.3 (2)
C6—C5—H5	119.3	C12—C13—H13	120.9
C5—C6—C1	118.29 (17)	C14—C13—H13	120.9
C5—C6—C7	119.87 (16)	C13—C14—C9	121.12 (18)
C1—C6—C7	121.84 (17)	C13—C14—S1	129.24 (16)
N2—C7—C6	122.23 (15)	C9—C14—S1	109.64 (14)
N2—C7—H7	118.9	C8—N1—C9	110.86 (15)
C6—C7—H7	118.9	C7—N2—C8	121.47 (15)
N1—C8—N2	121.36 (16)	C1—O1—H1	109.5
N1—C8—S1	115.61 (14)	C14—S1—C8	88.44 (9)
			
O1—C1—C2—C3	179.13 (19)	C12—C13—C14—C9	0.1 (3)
C6—C1—C2—C3	−0.4 (3)	C12—C13—C14—S1	−179.36 (17)
C1—C2—C3—C4	−0.1 (3)	N1—C9—C14—C13	179.63 (19)
C2—C3—C4—C5	0.5 (3)	C10—C9—C14—C13	−0.1 (3)
C3—C4—C5—C6	−0.3 (3)	N1—C9—C14—S1	−0.8 (2)
C4—C5—C6—C1	−0.2 (3)	C10—C9—C14—S1	179.44 (15)
C4—C5—C6—C7	179.96 (18)	N2—C8—N1—C9	179.65 (15)
O1—C1—C6—C5	−178.96 (17)	S1—C8—N1—C9	−1.0 (2)
C2—C1—C6—C5	0.5 (3)	C10—C9—N1—C8	−179.13 (18)
O1—C1—C6—C7	0.9 (3)	C14—C9—N1—C8	1.2 (2)
C2—C1—C6—C7	−179.64 (18)	C6—C7—N2—C8	−179.39 (16)
C5—C6—C7—N2	−179.38 (17)	N1—C8—N2—C7	−179.05 (17)
C1—C6—C7—N2	0.8 (3)	S1—C8—N2—C7	1.6 (2)
N1—C9—C10—C11	−179.61 (19)	C13—C14—S1—C8	179.7 (2)
C14—C9—C10—C11	0.1 (3)	C9—C14—S1—C8	0.24 (14)
C9—C10—C11—C12	−0.1 (3)	N1—C8—S1—C14	0.43 (15)
C10—C11—C12—C13	0.0 (4)	N2—C8—S1—C14	179.80 (15)
C11—C12—C13—C14	0.0 (4)		

### Hydrogen-bond geometry (Å, °)

**Table:**

*D*—H···*A*	*D*—H	H···*A*	*D*···*A*	*D*—H···*A*
O1—H1···N2	0.82	1.88	2.6034 (19)	147
C7—H7···O1^i^	0.93	2.43	3.309 (2)	158
C2—H2···N1^ii^	0.93	2.68	3.593 (2)	167

Symmetry codes: (i) *x*−1/2, *y*, −*z*+1/2; (ii) −*x*+2, *y*+1/2, −*z*+1/2.
